# The Molecular Dynamics Study of Vacancy Formation During Solidification of Pure Metals

**DOI:** 10.1038/s41598-017-10662-x

**Published:** 2017-08-31

**Authors:** H. Y. Zhang, F. Liu, Y. Yang, D. Y. Sun

**Affiliations:** 1 Department of Physics, East China Normal University, Shanghai, 200062 China; 2 Physical Science and Technology College, Yangzhou University, Yangzhou, 225002 China

## Abstract

In order to understand the defect trapping during solidification in pure elements, we have performed molecular dynamics simulations on both aluminum and nickel. We find that vacancies are the dominant defects in the product crystals for both metals. For slight undercooling, the vacancy concentration strongly depends on the growth velocity, rather than the growth orientations, and there is an approximately linear relationship between the growth velocity and vacancy concentration. However, for deep undercooling, the vacancy concentration shows a remarkable anisotropy between (100) and (110) orientations. Based on the competition between atomic diffusion and growth, a possible mechanism for vacancy trapping is suggested.

## Introduction

In the last couple of decades, computer simulations have been extensively applied on the solidification of metals^1, 2^. Up to date, the significant progress in understanding the solidification kinetics has been achieved on pure metals^3–36^ and a few binary alloys^37–41^. Despite the advances, one of the final pieces, namely the defect trapping during solidification, remains less investigated^42–51^. There are at least two reasons making this topic significant. First, the defect concentration may play an important role in determining the quality of the final solidification product. Second, the quantitative understanding of defect trapping continues to pose a major theoretical challenge.

There have been a few molecular dynamics (MD) studies on defect or solute trapping during solidification. Most of these studies have focused on alloys. For example, Zheng *et al*. have reported the obvious defect trapping including the formation of both antisite and vacancy in the crystallization of NiAl alloy^39^. Kramer, Mendelev and Napolitano have investigated the defect generation in the rapid solidification of the Zr_2_Cu compound^40^. Yang *et al*. have studied the solute trapping behavior for a Lennard-Jones model alloy^41^. However, the defect trapping in solidification process of pure elements is still less explored. The atomic mechanism of defect trapping, as well as its dependence on growth velocities and/or undercooling (defining as Δ*T* = *T*_*M*_ − *T*, *T*_*M*_ is the melting point) remains unclear. In this study, we take Ni and Al as typical examples to investigate the defect trapping behaviors during the solidification process.

Based on the current calculations, it is interesting to find that, only one type of point defects, namely the vacancy, is observed. The vacancy concentration not only depends on interfacial velocities but also depends on growth orientations at deep undercooling. However, at slight undercooling, the vacancy concentration seems to be only related to growth velocities.

## Results and Discussions

**Figure 1:**
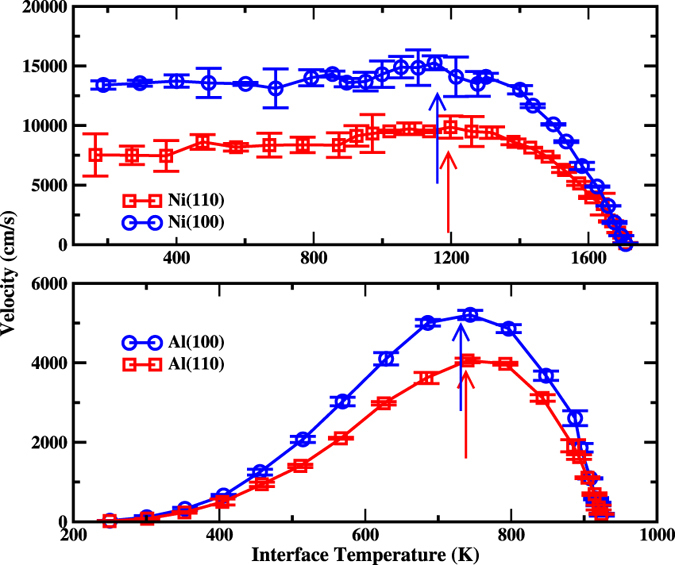
The crystal-melt interface velocity as a function of temperature for Ni (upper panel) and Al (lower panel). The arrow marks the temperature (T*), at which the interfacial velocity reaches the maximum.

Figure 1 plots the interfacial velocity as a function of temperature for both Ni (upper panel) and Al (lower panel). The error bars in interface velocities denote the standard errors in the mean value of interface velocity, obtained from the variance of the interface velocity derived separately from each of independent simulations for a given temperature. The interface velocities obey a linear relationship as a function of temperature in the range of small undercooling (Δ*T*). The constant of proportionality between interface velocities and temperature is the kinetic coefficient (*μ*), which characterizes the crystal-melt interfacial mobility. The present results for *μ* are listed in Table 1, which are in good agreement with the previous results^29, 31^.

**Table 1:** Values for the kinetic coefficient (*μ*) along (100) and (110) growth orientations of Ni and Al.

	Orientation	*μ*(*ms* ^−1^ *K* ^−1^) (refs 29, 31)	*μ*(*ms* ^−1^ *K* ^−1^) (This work)	T*(*K*)
Ni	(100)	0.71	0.66 ± 0.11	~1160
Ni	(110)	0.50	0.44 ± 0.04	~1192
Al	(100)	0.68	0.60 ± 0.23	~730
Al	(110)	0.59	0.48 ± 0.11	~738

The last row lists the temperature (*T* ^*^), at which the interfacial velocity reaches the maximum.

When the systems are cooled further, the interface velocity continues to increase until it reaches a maximum. A temperature (*T* ^*^), at which the interfacial velocity reaches the maximum, is defined. *T* ^*^ is indicated by arrows in Fig. 1, also listed in Table 1. It is interesting to find that, the growth velocity shows significant different temperature dependence between *T* > *T* ^*^ and *T* < *T* ^*^. For *T* > *T* ^*^, the growth velocity monotonically increases with the decrease of temperatures, while it keeps no increase as the undercooling decreasing at *T* < *T* ^*^. The velocity-temperature curves for both Ni and Al, either (110) or (100) orientations, are similar at *T* > *T* ^*^. When the temperature is below *T* ^*^, the velocity-temperature curve becomes much different between Ni and Al. Namely, with the decrease of temperature the velocity almost keeps constant for Ni, while for Al the velocity begins to decrease dramatically and decline to zero around 200 K. In the whole range of temperatures, the growth velocity of (100) orientation is always larger than that of (110) orientation for both Al and Ni. Our results are consistent with the general trends found in metals so far, and the velocity-temperature curve of Al and Ni represents two typical solidification processes in pure metals^26, 30, 36^.

The significant difference between Ni and Al at *T* < *T* ^*^ mentioned above may stem from the structural character of liquids. It has been found that, liquid Al with the current potential is more ordered than liquid Ni^31, 32^. However, the difference could be complicated and related to many factors. Indeed the growth velocity-temperature curve of Al and Ni is not isolated incident, which is also found in other metals^26, 30, 36^. Since our interests in current work is the defect trapping during the solidification, the issue regarding the growth mechanism and anisotropy will not be discussed further.

**Figure 2:**
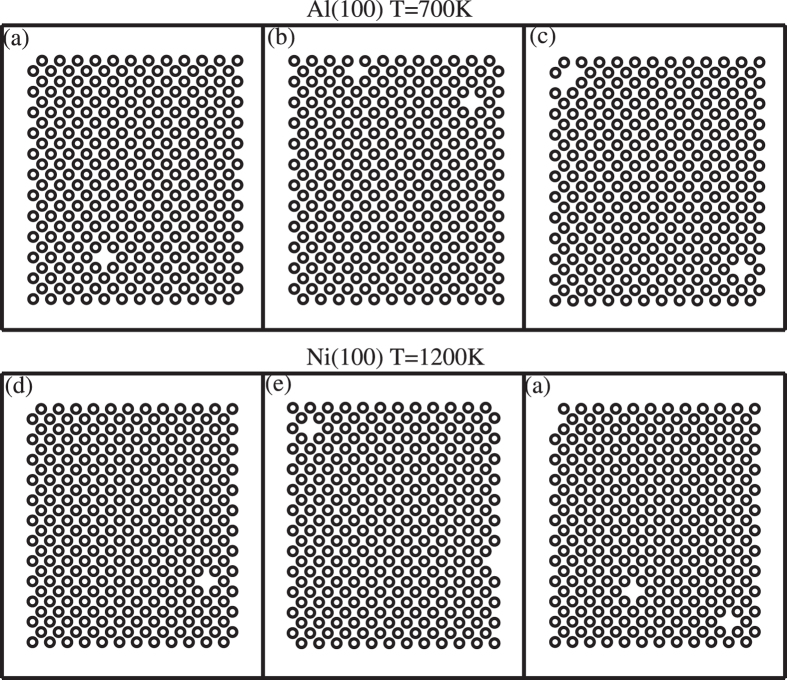
Snapshots of a few selected crystal planes after the solidification for Al(100) orientation (upper panel) and Ni(100) orientation (lower panel).

The point defects are observed at all the studied systems and temperatures. The dominated defect is the vacancy, but free of the interstitial. Even at the deep undercooling, only vacancies are observed. Figure 2 presents a few snapshots of lattice plane in the product crystal. We find that most vacancies are isolated. Only in a few cases, a pair of vacancies are formed (see Fig. 2(c)) but with an extremely low probability. Few vacancy clusters (including more than three vacancies) are observed.

**Figure 3:**
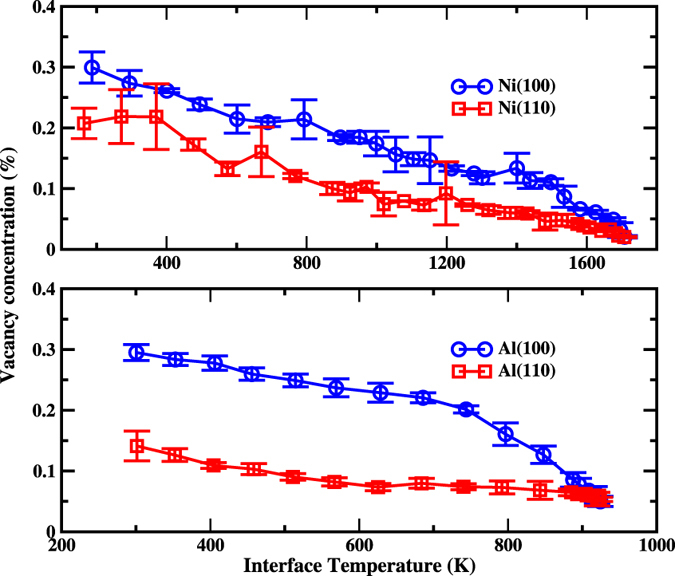
The concentration of vacancies in product crystal of Ni (upper panel) and Al (lower panel) as a function of interfacial temperatures.

The vacancy concentration as the function of interface temperature is depicted in Fig. 3. A few features can be seen from this figure. (1) Vacancy concentrations increase with the increase of undercooling in the whole range of temperature, which is much different from the growth velocity (Fig. 1). (2) A prominent anisotropy between (100) and (110) orientations for both metals is observed, and the vacancy concentration for (100) orientation is always larger than that of (110) orientation at the same temperature. (3) The vacancy generation is dependent on materials. The concentration of vacancies in Al is higher than that in Ni.

From Fig. 3, we can find another interesting feature. The concentration-temperature curves can be roughly viewed as two straight lines meeting at *T* ^*^. The slope of the straight line for *T* > *T* ^*^ is slightly larger than that for *T* < *T* ^*^. This feature is much evident in Al(100) orientations. As mentioned above, around *T* ^*^, the growth mechanism may change. The current results indicate that the growth mechanism could affect the disorder trapping. In turn, the disorder trapping may also affect growth mechanism. Although how the growth affecting the generation of vacancies remain unclear (we have proposed a possible mechanism in current work (see below)), the effect of vacancies on the growth is understandable, at least partially. With the increase of the vacancy concentration in solid phases, the free energy difference between solid and liquid will decrease, thus the driving force for the solidification (the free energy difference between solid and liquid phase) will be reduced accordingly.

Thermodynamically, point defects should always exist in a crystal above zero temperature. According to the data shown in Fig. 3, we could safely attribute the generation of vacancies mainly to the the rapid solidification of liquid, rather than the equilibrium thermodynamics effect. At least four facts support this interpretation. First, one can see that the vacancy concentration increases with the decrease of temperatures. If the vacancy concentrations correspond to thermodynamics equilibrium concentration of vacancies (TECV), it will show the opposite behavior, *i*.*e*., decreasing concentration with decreasing temperature. Second, the existence of the remarkable anisotropy indicates non-TECV character, since at thermodynamics equilibrium, the concentration of vacancies should not be correlated to growth directions. Third, according to the formation energy of vacancies in Al (0.69 eV)^52^ and Ni (1.63 eV)^53^, TECV of Al should be much higher than that of Ni at the same temperature. However as shown in Fig. 3, the vacancy concentrations between Al and Ni do not show much difference at the same temperature. At last, the vacancy concentrations shown in Fig. 3 are significantly larger than TECV (0.0016% and 0.02% for Ni and Al at melting temperature, respectively) calculated basing on the formation energy quoted above.

**Figure 4:**
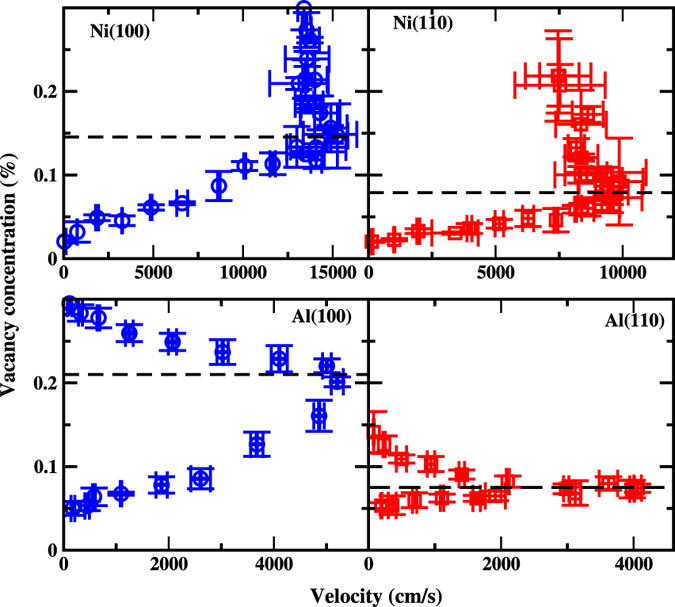
The concentration of vacancies in product crystal of Ni (upper panel) and Al (lower panel) as a function of interface velocities. The horizontal dash lines pass the maximum interfacial velocity. Below (above) the dash line, *T* > T* (*T* < T*).

In Fig. 4, we have shown vacancy concentrations via interface velocities for both Al and Ni. Although Fig. 4 is a re-packaging of the data in Figs 1 and 3, it does contain more information, which is hard to see from both Figs 1 and 3. One remarkable feature is that, the vacancy concentration is not single valued function of interfacial velocities. Data presented in Fig. 4 can be divided into two parts according to the temperature above or below *T* ^*^, which is indicated by a horizontal dash line. For the lower part (below the horizontal dashed line in Fig. 4), where the temperature is higher than *T* ^*^, the vacancy concentrations increase with the increase of interface velocities. In this part, there is no significant anisotropy between (100) and (110) orientations for small growth velocities, which is similar to the disorder trapping observed in NiAl alloy^39^. For another part (above the dashed line in Fig. 4), corresponding to the deep undercooling region (*T* < *T* ^*^), the opposite situation is observed, namely as the interface velocity increases, the anisotropy becomes more and more significant. The unexpected dependence of vacancy concentrations on interface velocities implies that, besides of growth velocities, there are other important facts strongly affecting the generation of vacancies. Future understanding of solidification process may require the knowledge of both defect trapping and growth mechanism.

Although the mechanism for vacancy trapping in pure metals remains unclear up to date, it is still possible to give a constructive discussion based on current results. As we have discussed above, for vacancy trapping, the kinetics rather than thermodynamics may play the leading role. In the viewpoint of kinetic effects, the crystal growth and atomic diffusion should be the major processes responding to the generation of vacancies. On the one hand, the crystallization process solidifies a disordered melt into an ordered crystal around the solid-liquid interface. This process can be imagined to *eliminate* liquid atoms at the interface. On the other hand, the diffusion process carries liquid atoms into the interface. Thus the competition between diffusion and growth could affect the production of vacancies. It is reasonable to speculate that the ratio between the diffusion flux and growth rate could play a role in the generation of vacancies. If the diffusion prevails, less vacancy will be expected. If diffusion does not prevail, then more vacancy should be expected.

To characterize the competition between atomic diffusion and growth process, we have defined a quantity $R(R=|\frac{{J}_{D}}{{J}_{G}}|)$, which describes the ratio between the diffusion flux (*J*_*D*_) and growth rate (*J*_*G*_). *J*_*D*_ is defined as the number of atoms passing the solid-liquid interface per unit time. According to Fick’s first law, we have, *J*_*D*_ = −*D*Δ*n*, where *D* and Δ*n* are the diffusion constant and atomic density gradient, respectively. ${\rm{\Delta }}n \sim \frac{{\rho }_{s}-{\rho }_{l}}{d}$, where *ρ*_*s*_ and *ρ*_*l*_ are the density of solid and liquid phase respectively, and *d* refers to the effective interfacial width. According to previous studies^12, 54^, an effective interfacial width can be approximately as three atomic layers, irrespective of the crystal orientation. In current work, *d* is taken as three times interfacial spacing. Similarly, we can define a growth rate, which characterizes the number of atoms *eliminated* through the solid-liquid interface per unit time. Thus, *J*_*G*_ = *V*(*ρ*_*s*_ − *ρ*_*l*_), where *V* is the interfacial velocity. Finally, we have $R=|\frac{{J}_{D}}{{J}_{G}}|=|\frac{D}{Vd}|$.

**Figure 5:**
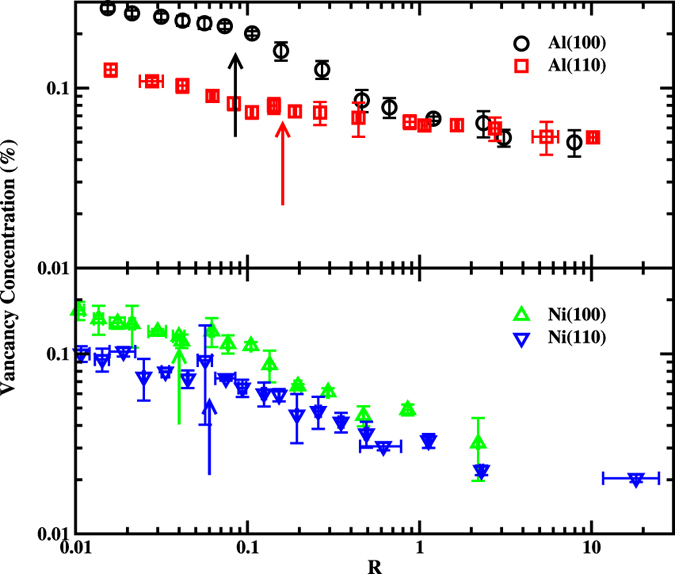
The concentration of vacancies as a function of *R* (the ratio between the diffusion flux and growth rate). The arrow marks T*.

As shown in Fig. 5, the positive correlation between the vacancy concentration and *R* can be found. From this figure, one can see that, with the increase of *R*, the vacancy concentration decreases monotonically. At large *R*, in which the atomic diffusion process is faster than the growth process, the concentration of vacancies is less. On the contrast, the growth rate surpasses diffusion flux when *R* is small, which is conducive to trap more vacancies. Similar to concentration-temperature curves, Fig. 5 can also be roughly viewed as two straight lines (in log-log plot) cross around *T* ^*^ (indicated by arrows). At *T* < *T* ^*^, the change of vacancy concentrations with *R* is slower than that at *T* > *T* ^*^. Although Al and Ni have much different growth behavior (see Fig. 1), the concentration-*R* curve shown in Fig. 5 is much similar to each other. As mentioned above, for the rapid solidification, a well-accepted theoretical or predictive model is still not available. The current study would shed light on both growth mechanism and disorder trapping.

At last, it is interesting to compare current results with that for B2 (CsCl prototype) ordered NiAl compound^39^. Similar to pure Ni and Al presented here, the vacancy is also one of the dominant defects in ordered NiAl compound. At a given growth velocity, the magnitude of the vacancy concentration in NiAl alloy is much larger than that of pure Al and Ni, which may stem from the different growth mechanism. What more, at small undercooling, similar to NiAl alloy, the vacancy concentration mainly depends on growth velocity, regardless of orientations.

In conclusion, our theoretical calculations demonstrate that, during the process of solidification, the notable vacancies is produced for both metals. For slight undercooling, the vacancy concentration is mainly dependent on the growth velocity, but weakly dependent on the interfacial orientation. For deep undercooling, the vacancy concentration does not solely depend on the growth velocity, but also shows a strong anisotropy behavior. Finally, based on the ratio between the diffusion flux and growth rate, a possible mechanism for the vacancy generation is also proposed. The present results could prompt people to re-evaluate the current model for solidification based on the kinetic attachment, and re-check the role played by the atomic diffusion.

## Methods

The MD simulations are performed by LAMMPS (large-scale atomic/molecular massively parallel simulator) code^55^. All simulations take a time step of 1 fs. Temperature and pressure of systems are controlled by using a Nos*e*′-Hoover thermostat^56, 57^ and Parrinello-Rahman barostat^58, 59^, respectively. The pressure of the system is kept at ambient pressure (1 bar). The many-body potential developed by Foiles *et al*.^53^ and by Ercolessi *et al*.^52^ are adopted for Ni and Al, respectively. Both potentials have been used to investigate the solidification process previously^23, 29–31, 60, 61^.

In order to set up proper simulation cells, the lattice constant as a function of temperature is calculated. Then the melting temperature, determined by using the modified coexistence approach^23^, is estimated around 939 K and 1710 K for Al and Ni respectively, which is in good agreement with the previous results^23, 31^. The simulation cells are established by taking a rectangular crystal of fcc Ni and Al crystal. The z direction is perpendicular to the solid-liquid interface, while the x and y directions are parallel to the solid-liquid interface. Periodic boundary conditions are used along three directions. The initial system for solidification simulations roughly containing 20% crystal and 80% liquid is equilibrated at melting temperatures. In order to guarantee the reliability of simulation results, the system has been set to have large cross-sectional area (*A* = *L*_*x*_ × *L*_*y*_) and very long length in z direction (*L*_*z*_). Two orientations, fcc (100) and fcc (110) of Ni and Al are considered in current studies. A 12 × 12 × 76 supercell including about 80640 atoms and a 8 × 12 × 84 supercell containing around 75264 atoms are used for (100) and (110) orientations, respectively.

**Figure 6:**
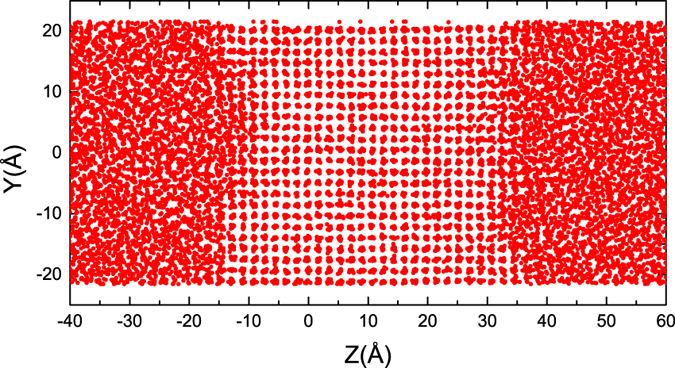
A snapshot of an initial equilibrium state used for subsequent solidification simulations (atoms only around solid-liquid interfaces are shown). Note, there are two crystal-melt interfaces in one periodic simulation cell.

The solidification simulations are performed under *NP*_*z*_ *AT* ensemble, the total number of atoms (*N*) and the cross-sectional area (*A*) are fixed, the length of the simulation cell perpendicular to the interfaces (*L*_*z*_) is dynamic to maintain constant pressure normal to the interface^22^. Since the length of systems in current calculations is much longer (about 60 nanometers), the self-interaction among the periodic images of interfaces can be safely neglected according to the previous studies. Non-equilibrium MD simulations start from the initial two phase coexistence configurations at *T*_*M*_, run under *T* < *T*_*M*_ with *A* being scaled to let crystal structure matching pre-calculated lattice constant at the given *T*. Figure 6 shows a snapshot illustrating the two-phase equilibrium state of the system, used as a starting point for subsequent non-equilibrium MD solidification simulations. To ensure reliable statistics, a few independent simulations are carried out at each undercooling. To track interfacial positions, an order parameter is calculated over narrow bins oriented parallel to the solid-liquid interface, and analyzed as a function of distance along the interfacial normal. The interfacial position thus is defined as the largest jump in order parameter. The order parameter is defined as, ${\varphi }_{i}=\mathrm{(1/}{N}_{n}){{\sum }_{i}|{r}_{ij}-{r}_{ij}^{ideal}|}^{2}$, where the sum extends over the *N*_*n*_ nearest neighbors, *r*_*ij*_ is the vector connecting sites *i* and *j* and ${r}_{ij}^{ideal}$ is the corresponding vector in an ideal crystal. For fcc crystal, *N*_*n*_ = 12. More details about the order parameter and the determination of interfacial position are referred to the work of Hoyt *et al*.^62^ and Sun *et al*.^22^.

Recent studies^29, 31, 34, 39, 41^ have shown that during non-equilibrium molecular dynamics simulations of solidification, the generation of latent heat at the moving solid-liquid boundary could lead to the formation of large temperature gradients around solid-liquid interfaces, which results in the interfacial temperature being different from the thermostat temperature. To obtain the true interface temperature, the temperature profiles along the interfacial normal is first calculated. Then the interface temperature is obtained by averaging the local temperature around the interface. In the current work, unless otherwise specified, the temperature quoted refers to the interfacial temperature, which is determined by using the method described in refs 29, 34.

**Figure 7:**
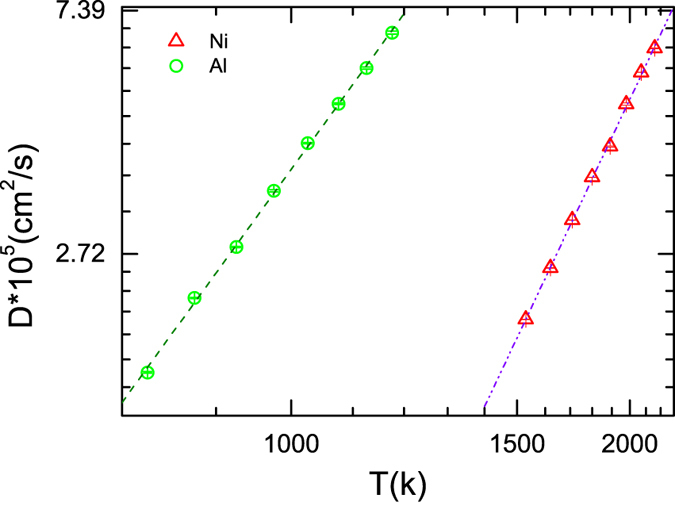
Diffusion constants versus temperature for Al (green circles) and Ni (red triangles), where the y-axis is logarithmic and the x-axis is reciprocal temperature. The dotted-dashed lines (Ni) and dashed lines (Al) are the Arrhenius fit to the data.

The temperature dependence of diffusion constant (*D*) of liquid Ni and Al is calculated. More specifically, a MD simulation is carried out on pure liquids at each temperature. For a given temperature, the mean-squared displacement (MSD) over each atom is calculated. The diffusion constant is directly calculated from the slope of the time evolution function of MSD. Figure 7 presents the plot of diffusion constant versus *T*, which can be well fitted by Arrhenius equation, namely *D* = *D*_0_exp(−*Q*/*k*_*B*_ *T*). Here *k*_*B*_, *Q* and *T* are Boltzmann constant, the activation energy for diffusions and the temperature, respectively. And *D*_0_ is a constant. By fitting to Arrhenius equation, we have obtained, *D*_0_ = 73.7 ± 1.1 × 10^9^ *m* ^2^/*s* (Al) and 59 ± 1 × 10^9^ *m* ^2^/*s* (Ni), *Q* = 0.315 ± 0.008 *eV* (Al) and 0.510 ± 0.007 *eV* (Ni). By assuming the diffusion holding the same activation energy throughout the whole temperature region, the diffusion constants of liquid can be extrapolated by Arrhenius equation.

To calculate the defect concentration in the product crystal, we follow the method used previously^39^. At the end of solidification, the product crystal is quenched to zero temperature using a steepest-descent algorithm to relax the atoms to their nearest local minimum. After this quenching, the atoms in the crystalline regions could be unambiguously assigned to the ideal crystal position. Then the real number of atoms in each crystal plane is counted, and defect concentrations are calculated. For each solidification process, before steady-state growth is observed to set in, the system undergoes a transient behavior with an initial period of approximately several hundreds picosecond (ps), which is a common feature in the simulation of solidification^23^. All the data and analysis are carried out on the crystal produced during steady-state growth.

### Data Availability Statement

All relevant data are within the paper.
